# Adult-onset xanthogranuloma: A rare variant with uncommon clinical presentation and dermatological challenges

**DOI:** 10.1016/j.jdcr.2026.03.051

**Published:** 2026-03-30

**Authors:** Milana E. Stein, Raj H. Patel, Emory K. Schmidt, Steven Cooper Heard

**Affiliations:** a New York Institute of Technology College of Osteopathic Medicine, Old Westbury, New York; b Department of Dermatology, HCA Healthcare/USF Morsani College of Medicine GME: HCA Florida Largo Hospital, Largo, Florida; c College of Health Sciences, University of South Florida, Tampa, Florida; d Dermatology & Skin Surgery, Shreveport, Louisiana

**Keywords:** adult orbital xanthogranulomatous disease, adult-onset xanthogranuloma, non-Langerhans cell histiocytosis, periorbital plaques, xanthogranulomatous inflammation

## Introduction

Adult-onset xanthogranuloma (AOX) is the rarest subtype within the spectrum of adult orbital xanthogranulomatous diseases (AOXGD), a group of non-Langerhans cell histiocytoses characterized by xanthogranulomatous infiltration of periocular and orbital tissues.^1^ Unlike other AOXGDs such as Erheim-Chester disease, necrobiotic xanthogranuloma, and adult-onset asthma with periocular xanthogranuloma, AOX is typically localized, self-limited, and lacks systemic involvement.^2^ Clinically, AOX often presents as yellow-orange periocular plaques or nodules and is considered benign.^3^ However, progressive orbital involvement may lead to functional and ophthalmologic complications if diagnosis is delayed. Due to its rarity, particularly with extracutaneous or distant cutaneous involvement, AOX is not as recognized in dermatologic practice. We report an unusual case of AOX presenting with bilateral periorbital plaques and a large indurated nodular lesion on the left upper arm, demonstrating the diagnostic considerations, histopathologic features, and therapeutic response.

## Case report

**Fig 1:**
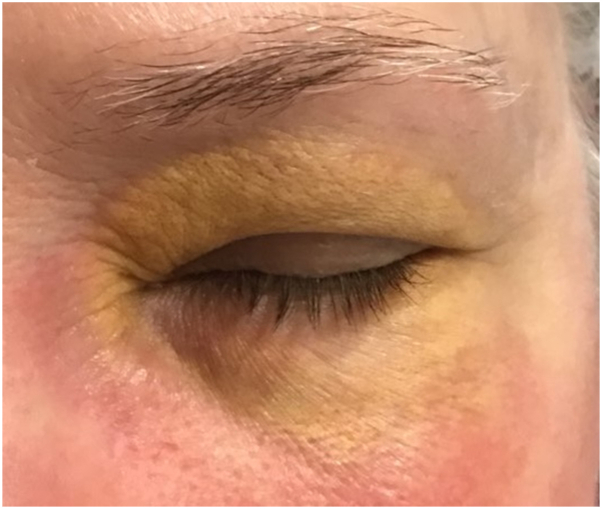
Clinical photographs of patient’s periorbital region presenting yellowish-red plaques bilaterally.

**Fig 2:**
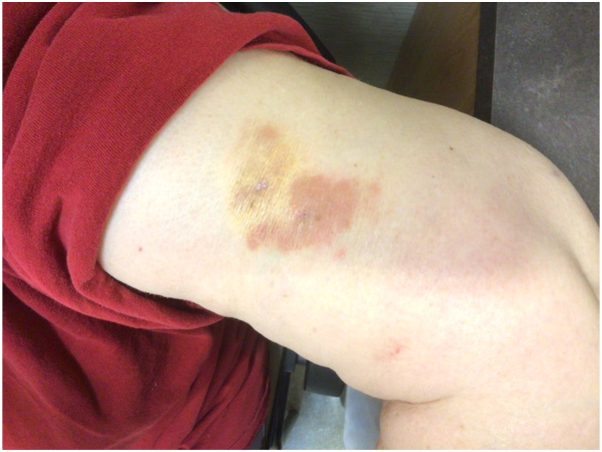
Clinical photographs of the patient’s left upper arm demonstrating a 4 cm indurated nodular mass with a peau d’orange surface.

A 72-year-old female presented with asymptomatic, yellowish-red plaques involving the bilateral periorbital region (Fig 1). Physical examination additionally revealed a firm, indurated 4 cm nodular mass on the left upper arm with a peau d’orange surface (Fig 2). The patient denied constitutional symptoms, visual changes, asthma, or a prior history of hematologic or systemic inflammatory disease.

**Fig 3:**
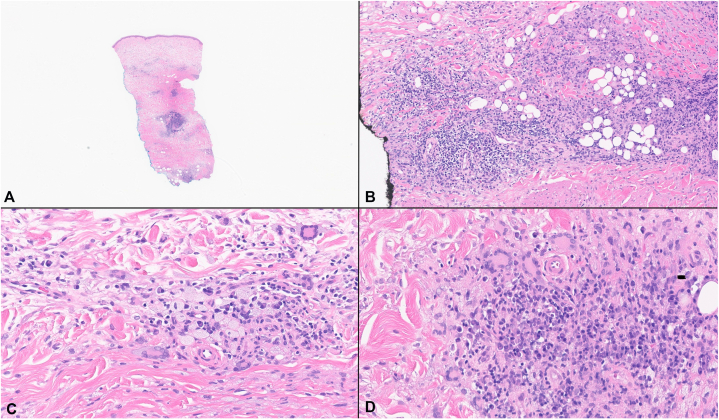
Histopathology of the left upper arm punch biopsy. Hematoxylin and eosin-stained sections demonstrate a dense superficial and deep dermal inflammatory infiltrate composed predominantly of lipidized (xanthomatous) histiocytes with scattered Touton-like giant cells, admixed plasma cells, eosinophils, and lymphocytes arranged in aggregates. The infiltrate extends into the superficial subcutis. **A,** 40×; **B-D,** 100×.

A punch biopsy of the left upper arm lesion was performed. Histopathologic examination demonstrated a dense superficial and deep dermal cellular infiltrate, extending into the superficial subcutis. The infiltrate was composed of lipid-laden xanthoma cells, Touton-like giant cells, plasma cells, eosinophils, and lymphocytes arranged in aggregates (Fig 3). Immunohistochemical staining showed strong CD68 positivity within the histiocytic population, consistent with a non-Langerhans cell histiocytosis. Colloidal iron staining was negative for increased dermal mucin. These findings supported the diagnosis of AOX.

Given the known association of certain xanthogranulomatous disorders with hematologic malignancies and proteinemias, the patient underwent evaluation by hematology/oncology. Comprehensive laboratory testing revealed no evidence of multiple myeloma, lymphoproliferative disease, hematologic abnormalities, or paraproteinemia. There was no clinical history suggestive of asthma or systemic involvement.

Oral corticosteroid therapy was initiated 2 weeks after biopsy confirmation of AOX. The patient was treated with oral prednisone at a dose of 20 mg once daily for a total of 30 days, resulting in marked improvement of the lesions. This dose was subsequently tapered in a stepwise fashion as follows: 20 mg and 10 mg on alternating days for 2 weeks, 20 mg and 5 mg on alternating days for 2 weeks, 10 mg every other day for 2 weeks, and 5 mg every other day for 3 weeks, until clinical resolution and disease control was achieved. The upper arm lesion was additionally treated with fluocinonide 0.05% cream twice daily for 2 weeks, and then used intermittently in 2-week on/off cycles as needed. Ongoing clinical follow-up was arranged to monitor for recurrence or progression.

## Discussion

Adult-onset xanthogranuloma is a rare and poorly understood subtype of AOXGD. Unlike necrobiotic xanthrogranuloma and Erdehim-Chester disease, AOX is typically confined to periocular tissues and lacks systemic manifestations or strong associations with paraproteinemias.^4^ Histopathologically, AOX is characterized by mixed inflammatory infiltrate rich in foamy histiocytes and Touton giant cells, with CD68 positivity and absence of Langerhans cell markers.^1^

The presentation of AOX with a large, distant cutaneous nodular lesion, as seen in this patient’s upper arm, is uncommon and sparsely reported in the literature.^3^ This atypical distribution may pose a diagnostic challenge and broaden the differential to include xanthomas, necrobiotic xanthogranuloma, sarcoidosis, and other histolytic disorders. Thorough clinicopathologic correlation and systemic evaluation are essential.

AOX most commonly presents with isolated pre-septal and anterior orbital lesions that may be self-limited, rendering it the rarest of the 4 entities.^2^ Management of AOX is not standardized due to its rarity. Reported treatment modalities include surgical excision, oral, intralesional or systemic corticosteroids, immunosuppressive agents, chemotherapy, and radiation therapy, depending on disease severity and extent.^5^ While oral corticosteroids are commonly used as initial therapy, treatment failure or disease recurrence have been reported.^2^ This necessitates prolonged or maintenance systemic corticosteroid therapy to achieve sustained disease control.^2^ In contrast, our patient demonstrated a marked clinical response to oral corticosteroids without evidence of early recurrence. Early recognition is particularly important given the potential for orbital involvement, which may result in visual impairment if left untreated.^6^

## Conclusion

This case highlights a rare presentation of adult-onset xanthogranuloma with periocular and distant cutaneous involvement. Despite the unusual distribution that included a large distant cutaneous nodular lesion, histopathologic evaluations revealed no features suggestive of other AOXGD. Cumulative findings support the diagnosis of isolated AOX, a rare entity within the AOXGD spectrum. Further expanding the recognition of this AOXGD and its characteristic histopathologic features is essential to ensure accurate diagnosis, appropriate systemic evaluation, and timely treatment. Early intervention may help prevent disease progression and reduce the risk of ophthalmologic complications.

## Conflicts of interest

None disclosed.
